# Anterior femoral offset is a flawed measurement of patellofemoral overstuffing

**DOI:** 10.1007/s00402-024-05662-2

**Published:** 2024-12-12

**Authors:** Antonio Klasan, Thomas Jan Heyse, Alexander Johannes Nedopil

**Affiliations:** 1 AUVA UKH Steiermark, AUVA UKH Steiermark, Göstinger Str. 24, 8020 Graz, Austria; 2 Johannes Kepler University Linz, Linz, Austria; 3 Red Cross Hospital, Frankfurt, Germany; 4 Adventist Health Memorial Hospital, Lodi, CA USA; 5 University of Würzburg, Würzburg, Germany

**Keywords:** Patellofemoral joint, Overstuffing, Total knee arthroplasty, Flange, Anterior offset

## Abstract

**Background:**

Patellofemoral joint (PFJ) issues after total knee arthroplasty (TKA) are becoming a topic of interest once again. Due to the complex three-dimensional shape of the trochlea, various two-dimensional proxy measurements on plain X-rays have been described. One of these measurements is the anterior femoral offset (AFO). It is the distance between the anterior femoral cortex and the trochlea’s most anterior point (MAP) on a true lateral X-ray after TKA. Because the relationship between the trochlea’s MAP and its groove, which is the primary joint surface articulating with the patella, is unknown, the purpose of this study was to measure the distance between the MAP and the trochlear groove.

**Methods:**

After femoral component implantation, the surgeon identified the trochlea’s MAP and the trochlear groove in ten consecutive TKAs and measured their distance. Measurements were performed with a ruler on a true lateral photograph and with a radiographic marker on a lateral radiograph in four different knee flexion angles, according to a previously published protocol.

**Results:**

The trochlear groove had a mean distance from the MAP between 2.09 ± 0.15 and 5.50 ± 0.17 mm, depending on the position. In no case is the trochlear groove visible on a true lateral view.

**Conclusion:**

On a true lateral X-ray, the trochlea’s MAP omits the trochlear groove. Because the patella primarily articulates with the trochlear groove and because the relationship between the MAP and the trochlear groove is variable depending on the knee flexion angle, any conclusions regarding overstuffing based on a postoperative lateral knee X-ray are flawed.

**Supplementary Information:**

The online version contains supplementary material available at 10.1007/s00402-024-05662-2.

## Introduction

There is an undeniable rise in the interest in the patellofemoral joint (PFJ) and how it is affected after total knee arthroplasty (TKA) as we are plateauing with outcomes and need to look closer at how and even if to operate [1]. On one side, alignment of the prosthetic trochlea is affected by the alignment strategy and implant design [2]. Newer designs specifically target the PFJ, aiming to be more “friendly” towards the second joint in the knee [3].

On the other side, the rotation, position and size of the femoral component in the sagittal plane also affect the PFJ [4], as is the case with the native femoral torsion [5], irrespective of the patella position [6].

Upsizing or anteriorizing of the component might replace more bone and cartilage with metal, thus creating over-stuffing [7]. Conversely, downsizing or posteriorizing of the femoral component creates under-stuffing [8]. Flexing and extending of the component alters patellofemoral biomechanics [9].

Altering the trochlear position in the sagittal plane greatly affects the forces needed by the quadriceps muscle and tendon [4].

The most commonly used measurement of under/overstuffing is anterior femoral offset (AFO) or patellofemoral joint offset (PFJO), which is computed on a true lateral x-ray of the knee. On postoperative x-rays, a line through the anterior femoral cortex is drawn and a second line tangent to the most anterior point of the prosthetic trochlea visualized on the x-ray, is drawn, parallel to the first line with the distance between these two lines representing AFO/PFJO [10].

Whereas preoperative lateral x-rays allow for visualisation of the lateral facette and of the trochlear groove, this is not the case on postoperative x-rays.

The purpose of this study was to investigate the distance between the most anterior point of the implant, measured on lateral postoperative x-rays, to the trochlear groove of the prosthetic implant. We hypothesized that the trochlear groove is significantly deeper than the MAP measured on x-rays and cannot be visualized under any circumstances.

## Methods

### Patients

This is a prospective study of 10 consecutive patients undergoing TKA in a single center by a single surgeon (A.K.), between 12/2023 and 02/2024. The center has a prospective Ethics Board approval 17/2021 for robotic assisted surgery (RAS) TKA. In all cases RAS-TKA was performed (MAKO Rio, Stryker, Kalamazoo, MI, U.S.) using Triathlon (Stryker) implants. All cases in the present study were performed using a medial parapatellar approach and functional alignment strategy [11]. We recorded age and gender, as well as coronal phenotype [12].

### Measurement technique

**Fig. 1:**
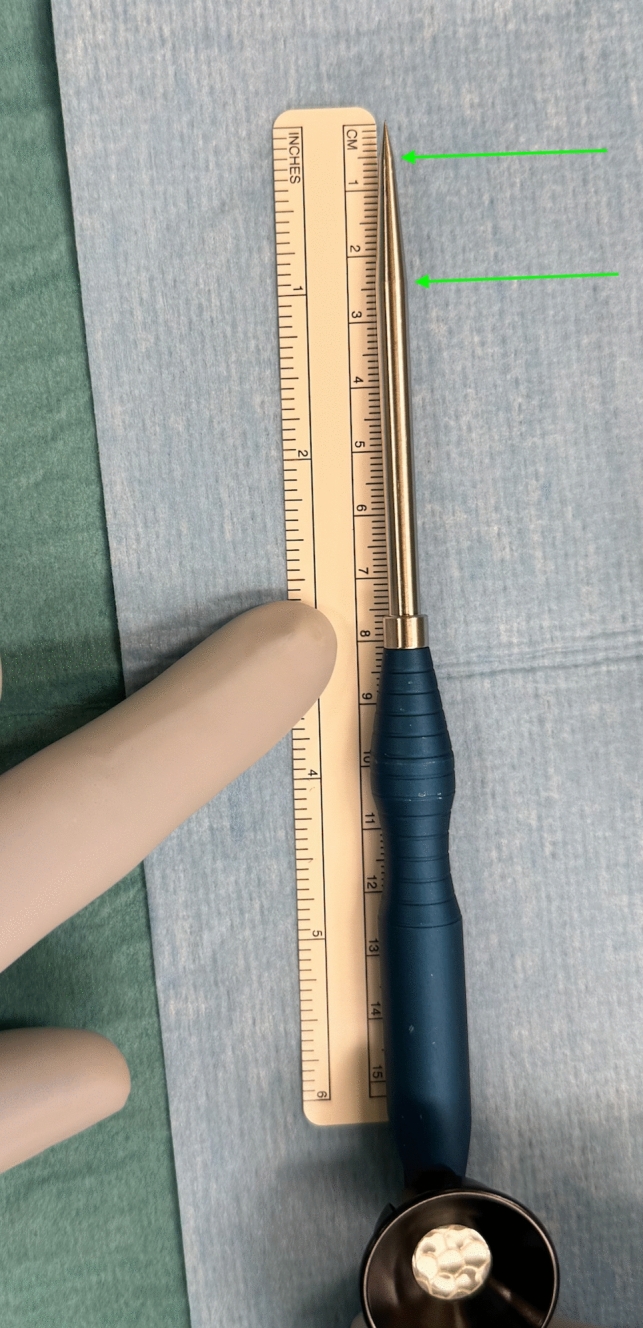
The sharp probe used to demonstrate the trochlear position on lateral x ray view

**Fig. 2:**
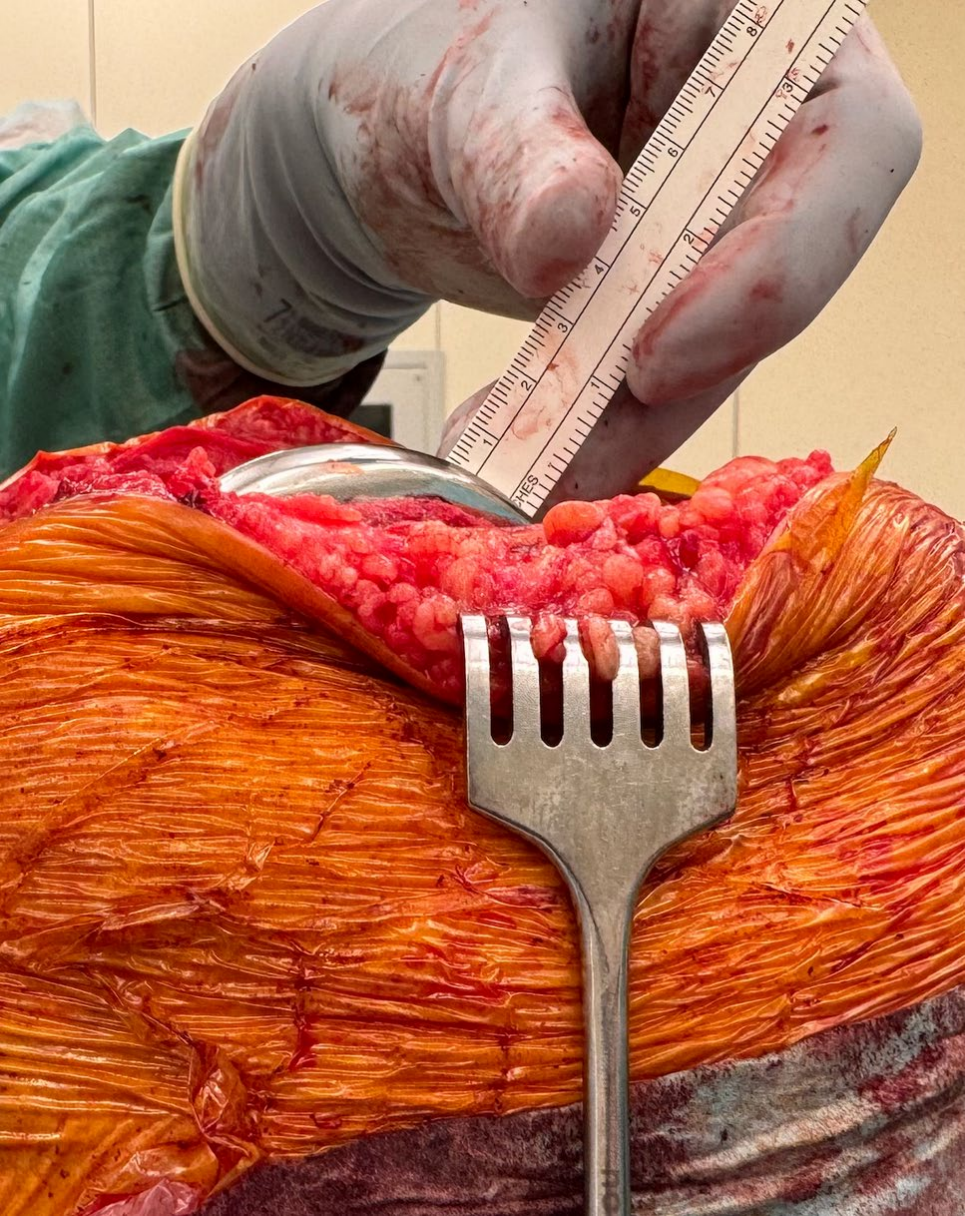
Measurement technique using a measurement strip on a direct lateral view

Prior to wound closure, a blue cartilage Probe (Stryker), Fig. 1, and a paper ruler, with the starting point cut at 0 mm, were used to measure and visualize the trochlear groove of the implant in a true lateral photographic and radiographic view at the positions defined as per Kafelov et al. [13], with respect to the most anterior point. The probe has a widening at 5 mm and at 14 mm, Figs. 1, 2.

**Fig. 3:**
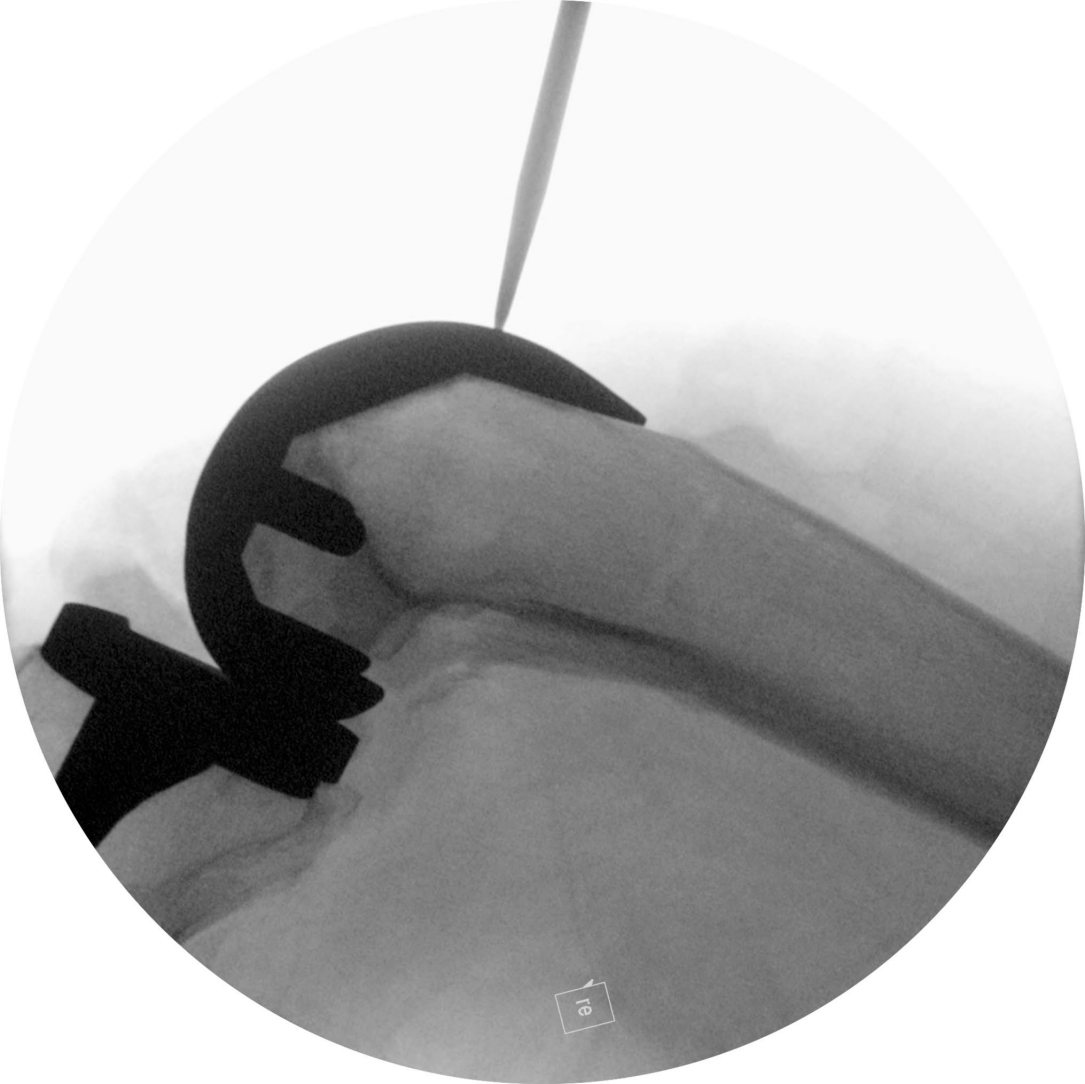
Lateral x-ray view demonstrating the sharp probe’s tip omitted by the lateral facette

Then, a lateral x-ray was performed in the same manner using the blue probe, Fig. 3.

Depth measurements photographs were evaluated by two investigators (A.K., A.J.N.) twice, with 2 weeks between the readings. Each measurement at each position is the mean value of the 4 measurement attempts.

### Statistical analysis

For each position, mean depth (± standard deviation) of trochlear groove from the most anterior point for was calculated. Inter- und intraobserver agreement was evaluated using intraclass correlation coefficient (ICC). An a priori power analysis was performed. Using Alpha of 0.05 and Beta 0.8, for a difference of 2 ± 1 mm, 8 patients were needed to demonstrate a difference. Statistical analysis was performed using SPSS 29 (IBM, Armonk, NY, U.S.). Statistical significance was set at p < 0.05.

## Results

### Patients

Mean patient age was 66.7 ± 8.4 years, mean BMI was 27.4 ± 6.5. Gender was evenly distributed. In terms of HKA, one patient was VAR_HKA_3°, three patients VAR_HKA_6°, four patients VAR_HKA_9° and two patients VAL_HKA_9°.

On all four positions, the mean difference between the deepest point of the trochlear groove and the femoral offset was > 2 mm, Table 1. ICC for intra- and interobserver measurements was 0.96 and 0.92, respectively.

**Table 1:** Mean measurement values

Position	1	2	3	4
Mean difference (± SD) mm	2.09 ± 0.15	3.14 ± 0.20	5.50 ± 0.17	5.50 ± 0.14

## Discussion

The present study demonstrates the significant difference between the articulating surface of the trochlea, the groove, and the most anterior point of the lateral facette, the anterior offset, after TKA.

With the increasing interest in the patellofemoral joint issues after TKA, various measurements of the trochlea can be performed. Due to the complexity of the measurement of the three-dimensional trochlear shape, various proxy measurements in two-dimensions are being used. One such common measurement is anterior femoral offset (AFO) or patellofemoral joint offset (PFJO). A recent systematic review demonstrated multiple studies using this measurement [7]. The clinical role of this measurement in the literature is however, debated. Pierson et al. demonstrated no correlation to clinical outcomes in 1100 TKAs [10], Matz et al. in 970 TKAs [14] and Beldman in 193 TKAs [15]. On the other hand, Kemp et. al demonstrated negative association between outcome and increased anterior offset in TKA [16]. Raba et al. demonstrated negative outcomes with anterior overstuffing in an unlinked medial unicompartimental and patellofemoral arthroplasty [17]. Isolated patellofemoral arthroplasty, traditionally the worse performing partial knee arthroplasty, nowadays seems to be a viable alternative to TKA for patients with PFOA [18], confirming the importance of better understanding of this compartment.

Changes in patella implant thickness negatively affect flexion and PFJ kinematics, but after an increase of patellar thickness of at least 6 mm [19]. Whereas patella thickness measurements are easy to obtain pre- and postoperatively due to a clean visualization of the patella thickness on a true lateral view [7], this is not the case with the trochlear groove. Implant designs vary in trochlear shape and often demonstrate signs of dysplastic trochlea [2]. The 3D visualization of various implant trochleae performed by Itou et al. demonstrated that the lateral, central and medial anterior most prominent point vary between implant designs [20]. Itou et al. performed the measurement relatively proximally in the trochlea, where the patella would sit in extension, corresponding to position 1 described by Kafelev et al. [13] and in the present study. Due to the fact that the lateral most prominent point is always the most anterior both for Triathlon (Stryker), used in the present study, as is the case with all other implants [2, 20], the lateral implant facette radiographically omits the view of the central and medial most prominent point completely. For Triathlon, this difference is between 2 and 5 mm, depending on the position. Since the patella tracks primarily in the trochlear groove and towards but not on the most anterior point of the lateral facette, any conclusion with respect to the trochlear position based solely on lateral x-ray is impossible to make. Excessive internal rotation of the femoral component, which alters patellofemoral kinematics significantly and can lead to patella dislocation [21] is measured using transverse CT scans [22] and is practically undetectable on lateral x rays as the groove and medial facette remain radiographically omitted.

Several limitations need to be noted. Firstly, only one implant design was evaluated, however, all implants try to replicate the native trochlea by increasing the lateral facette [2, 20, 23]. Secondly, the measurements were performed in vivo using a paper measurement strip and a metal probe with small measurement errors being possible. Due to ethical considerations, prolonging the surgery was not allowed and a cadaveric study would provide more exact measurements, however, in no case would the trochlear groove match the anterior femoral offset measurement.

## Conclusion

The implant trochlear groove is radiologically omitted by the implant lateral facette on true lateral x rays after TKA. Since the lateral facette is not the primary joint surface corresponding to the patella in the patellofemoral joint, any conclusions based on lateral knee x-ray with regards to overstuffing are flawed.

## Supplementary Information

Below is the link to the electronic supplementary material.

Supplementary file1 (DOCX 12 KB)

Supplementary file2 (DOCX 12 KB)
