# Supplementary material for: Anterior femoral offset is a flawed measurement of patellofemoral overstuffing
Source: Arch Orthop Trauma Surg. 2024 Dec 12;145(1):34. doi: 10.1007/s00402-024-05662-2 (PMC11638414; doi:10.1007/s00402-024-05662-2)
Supplement: Supplementary file 1 — Supplementary file1 (DOCX 12 KB) [file 402_2024_5662_MOESM1_ESM.docx]

Declarations

The authors did not receive support from any organization for the submitted work.

AK has been paid for presentations by Arthrex, FH Ortho and Implantcast. He is an associate editor for Journal of Knee Surgery and editorial board member of KSSTA and AOTS. TJH has been paid for presentations by Smith&Nephew. AJN receives royalties from Medacta, has been paid for presentations by Smith&Nephew and Microport. He is a consultant for Smith&Nephew, Medacta and Think Surgical.

The institution has an ongoing ethics board approval by the AUVA Ethics Board (17/2021). Informed consent was provided by the participants.
