# Supplementary material for: Anterior femoral offset is a flawed measurement of patellofemoral overstuffing
Source: Arch Orthop Trauma Surg. 2024 Dec 12;145(1):34. doi: 10.1007/s00402-024-05662-2 (PMC11638414; doi:10.1007/s00402-024-05662-2)
Supplement: Supplementary file 2 — Supplementary file2 (DOCX 12 KB) [file 402_2024_5662_MOESM2_ESM.docx]

Figure 1 – The sharp probe used to demonstrate the trochlear position on lateral x ray view.

Figure 2 – Measurement technique using a measurement strip on a direct lateral view.

Figure 3 – Lateral x-ray view demonstrating the sharp probe’s tip omitted by the lateral facette.
